# Remote Ischemic Conditioning as an Adjunct to Primary Percutaneous Coronary Intervention in ST-Elevation Myocardial Infarction: A Systematic Review and Meta-Analysis

**DOI:** 10.7759/cureus.109487

**Published:** 2026-05-23

**Authors:** Mohammed A Khormi, Hussain M Al Qibti, Maram M Fageehi, Hanan M Shawlan, Ahmad H Mathary, Ziyad S Alsaeedi, Sultan H Baghdadi, Mohammad H Alshahrani, Sultan A Alghamdi, Ziyad A Alfarsi, Saad F Aljuadi, Abdulrahim H Shammakhi

**Affiliations:** 1 Department of Family Medicine, Jazan Health Cluster, Jazan, SAU; 2 Department of Internal Medicine, King Fahad Central Hospital, Jazan, SAU; 3 Department of Internal Medicine, Prince Sultan Military Medical City, Riyadh, SAU; 4 College of Medicine, King Abdulaziz University, Jeddah, SAU; 5 Department of Emergency Medicine, Ahad Rufaidah General Hospital, Ahad Rufaidah, SAU; 6 College of Medicine, Prince Sattam bin Abdulaziz University, Al Kharj, SAU; 7 Faculty of Medicine, Jazan University, Jazan, SAU

**Keywords:** infarct size, left ventricular ejection fraction, meta-analysis, myocardial infarction, pci, primary percutaneous coronary intervention, randomized controlled trials, remote ischaemic conditioning, st-elevation myocardial infarction, systematic review

## Abstract

Remote ischaemic conditioning (RIC) is a non-invasive technique that applies brief, repeated cycles of limb ischaemia and reperfusion using a blood pressure cuff and has been investigated as an adjunct cardioprotective strategy during primary percutaneous coronary intervention (PPCI) in patients with ST-elevation myocardial infarction (STEMI). Despite encouraging experimental data, findings from randomized controlled trials (RCTs) have been inconsistent, and their overall impact on both functional and clinical outcomes remains uncertain. We performed a systematic review and meta-analysis of RCTs comparing RIC plus PPCI with PPCI alone in patients with STEMI.

A comprehensive search of PubMed, Cochrane Library, Scopus, and Web of Science was conducted up to March 2026. The primary endpoints were left ventricular ejection fraction (LVEF) and infarct size, while secondary endpoints included all-cause mortality, cardiovascular mortality, major adverse cardiovascular events (MACE), heart failure hospitalization, recurrent myocardial infarction (MI), and repeat revascularization. Data were pooled using a random-effects DerSimonian-Laird model, and outcomes were reported as mean difference (MD), risk ratio (RR), log risk ratio, and 95% confidence interval (CI). A total of 21 RCTs encompassing 8,392 patients were included.

Remote ischaemic conditioning showed a modest but statistically significant increase in LVEF (MD 1.23%, 95% CI 0.05-2.42; p = 0.04; I² = 0%) and a reduction in recurrent MI (log RR −0.24; 95% CI −0.48 to −0.01; p = 0.04; I² = 0%). No significant effect was observed on infarct size (MD −0.62; p = 0.22). Similarly, no differences were identified in all-cause mortality, cardiovascular mortality, MACE, heart failure hospitalization, or repeat revascularization. Sensitivity analyses confirmed the robustness of most outcomes, although exclusion of the CONDI-2/ERIC-PPCI trial influenced the significance of cardiovascular mortality and MACE.

Overall, in STEMI patients undergoing PPCI, adjunctive RIC may provide modest improvement in left ventricular function and reduction in recurrent MI, but it does not clearly reduce major clinical events. Current evidence does not support routine clinical use, and further well-designed stratified trials are required to identify patient groups who may benefit most and to optimize treatment protocols.

## Introduction and background

ST-elevation myocardial infarction (STEMI) continues to be a major global contributor to cardiovascular morbidity and mortality [1-8]. Although primary percutaneous coronary intervention (PPCI) represents the cornerstone of reperfusion therapy in acute myocardial infarction (MI), myocardial damage is not limited solely to the period of ischemia. A substantial proportion of injury occurs upon restoration of blood flow, a process known as ischaemia-reperfusion injury (IRI). This phenomenon plays a central role in determining final infarct size and is strongly associated with adverse ventricular remodeling, development of heart failure, and long-term mortality following STEMI [5-13].

Remote ischaemic conditioning (RIC) is a simple, non-invasive strategy that involves intermittent cycles of brief limb ischemia and reperfusion, commonly achieved using a blood pressure cuff. The intervention is thought to activate endogenous protective pathways that increase myocardial resistance to reperfusion-related injury [10-14]. Depending on the timing of application, RIC may be administered before reperfusion (preconditioning), during reperfusion (perconditioning), or after reperfusion (postconditioning), with each approach targeting distinct phases of myocardial injury [15-20].

Early randomized controlled trials (RCTs) investigating RIC as an adjunct to PPCI reported encouraging results, including reductions in infarct size and improvements in surrogate indicators of myocardial injury and left ventricular performance, particularly left ventricular ejection fraction (LVEF) [17-21]. These findings supported the underlying biological rationale and stimulated interest in RIC as a simple and inexpensive adjunct to standard reperfusion therapy.

However, later large multicenter RCTs failed to reproduce consistent benefits in infarct size or major clinical outcomes, creating uncertainty regarding its true therapeutic value [1-15]. This inconsistency has been attributed to differences in trial design, patient characteristics, timing, and protocol of intervention, concomitant therapies, and outcome assessment methods.

In light of these conflicting results, there remains a need for a comprehensive synthesis of randomized evidence to better define the overall effect of RIC in STEMI patients undergoing PPCI, particularly with respect to both functional measures, such as LVEF and infarct size, as well as clinically important outcomes, including mortality and heart failure events.

## Review

Methodology

This systematic review and meta-analysis was conducted in accordance with the Preferred Reporting Items for Systematic Reviews and Meta-Analyses (PRISMA) 2020 guidelines [22].

Literature Search and Screening

A comprehensive search was performed up to March 2026 across four electronic databases: PubMed, Cochrane Library, Scopus, and Web of Science (WOS). No date restrictions were applied. The search strategy combined Medical Subject Headings (MeSH) terms and free-text keywords using Boolean operators (Table 1 and Table 2).

**Table 1:** PICO framework defining eligibility criteria for study selection This table summarizes the Population, Intervention, Comparator, and Outcomes (PICO) framework used to define eligibility criteria for inclusion in this systematic review and meta-analysis of randomized controlled trials evaluating remote ischaemic conditioning (RIC) in patients with ST-elevation myocardial infarction (STEMI) undergoing primary percutaneous coronary intervention (PPCI).

Component	Description
Population (P)	Adults (≥18 years) with acute ST-elevation myocardial infarction (STEMI) undergoing primary percutaneous coronary intervention (PPCI)
Intervention (I)	Remote ischaemic conditioning (RIC), including remote ischaemic preconditioning, perconditioning, postconditioning, or repeated/chronic limb ischaemia-reperfusion cycles using a blood pressure cuff (≥200 mmHg or above systolic blood pressure)
Comparator (C)	Standard care PPCI, sham RIC, or placebo intervention without limb ischemia-reperfusion conditioning
Outcomes (O)	Primary: Left ventricular ejection fraction (LVEF), infarct size. Secondary: all-cause mortality, cardiovascular mortality, major adverse cardiovascular events (MACE), heart failure hospitalisation, recurrent myocardial infarction, repeat revascularisation
Study design	Randomized controlled trials (RCTs) only

**Table 2:** Literature search strategy and information sources This table outlines the systematic search strategy used to identify relevant studies. Four electronic databases (PubMed, Cochrane Library, Scopus, and Web of Science) were searched from inception to March 2026. The table details the number of records retrieved from each database, search terms applied, eligibility filters, and supplementary search methods used to ensure comprehensive study identification in accordance with PRISMA 2020 guidelines. Abbreviations: RIC, remote ischaemic conditioning; RIPC, remote ischaemic preconditioning; STEMI, ST-elevation myocardial infarction; PCI, percutaneous coronary intervention; PPCI, primary percutaneous coronary intervention; WOS, Web of Science; PRISMA, Preferred Reporting Items for Systematic Reviews and Meta-Analyses

Database	Records identified (n)	Search terms and filters applied
PubMed	184	RIC, RIPC, STEMI, myocardial infarction, PCI; no language restriction
Cochrane Library	227	RIC, remote ischaemic conditioning, myocardial infarction, PCI trials
Scopus	153	English-language human studies; original articles; RIC-related keywords
Web of Science (WOS)	327	English-language filters applied; RIC, STEMI, PCI, myocardial infarction
Total	891	Combined search strategy using MeSH terms and free-text keywords

Search terms covered remote ischaemic conditioning (RIC) and remote ischaemic preconditioning (RIPC), along with clinical conditions such as ST-elevation myocardial infarction (STEMI), non-ST-elevation myocardial infarction (NSTEMI), myocardial infarction (MI), and coronary artery disease (CAD), as well as procedural terms including percutaneous coronary intervention (PCI) and revascularisation. Filters were applied in Scopus and WOS for English-language human studies and original articles. No language restrictions were applied in PubMed or the Cochrane Library.

Reference lists of included studies and relevant reviews were manually screened to identify additional eligible studies. All records were imported into a reference manager, and duplicates were removed before screening. Two independent reviewers screened titles and abstracts, followed by full-text assessment of potentially eligible studies. Disagreements were resolved by consensus or adjudication by a third reviewer.

Eligibility Criteria

Studies were selected according to Population, Intervention, Comparator, and Outcomes (PICO) criteria [23]. The population included adults (≥18 years) with acute myocardial infarction undergoing PPCI. Paediatric studies were excluded.

The intervention was any form of RIC applied to a limb (upper or lower) using blood pressure cuff inflation at ≥200 mmHg or above systolic blood pressure. This included remote ischaemic preconditioning (RIPC), perconditioning, post-conditioning, repeated/chronic protocols, or combined approaches. Local ischaemic conditioning without a remote component was excluded.

Comparators included no RIC, sham RIC, or alternative reperfusion strategies. Primary outcomes were LVEF and infarct size. Secondary outcomes included all-cause mortality, cardiovascular mortality, major adverse cardiovascular events (MACE), heart failure (HF) hospitalisation, recurrent MI, and revascularisation. Only randomized controlled trials (RCTs) were included.

Data Extraction

Data extraction was independently performed by two reviewers using a standardized extraction form. Extracted variables included study design, setting, sample size, patient characteristics, intervention details (type, timing, cycles, inflation pressure, duration, and device), procedural variables, and comparator information. Any inconsistencies were resolved by consensus or third-party review.

Risk of Bias Assessment

Methodological quality was assessed using the Cochrane Risk of Bias 2 (RoB 2) tool, which evaluates randomization, deviations from intended intervention, missing outcome data, outcome measurement, and selective reporting. Assessments were conducted independently by two reviewers, with arbitration by a third reviewer when required [24].

Statistical Analysis

Continuous outcomes (LVEF and infarct size) were summarized using mean differences (MD) with 95% confidence intervals (CI). Binary outcomes (all-cause mortality, cardiovascular mortality, MACE, HF hospitalisation, recurrent MI, and revascularisation) were analysed using log risk ratios (log RR), which were converted to risk ratios (RR) with 95% CI.

A random-effects model using the DerSimonian-Laird method was applied to account for between-study heterogeneity. Heterogeneity was assessed using Cochran’s Q test (p < 0.10 for significance), I² statistics (low <25%, moderate 25-50%, high >50%), τ², and H² statistics. Statistical significance was defined as a two-sided p-value <0.05.

Leave-one-out sensitivity analyses were performed by sequentially excluding individual studies and recalculating pooled estimates. All analyses were conducted using Stata version 17.0 (StataCorp LLC, College Station, TX, USA), and forest plots were generated for presentation.

Results

Study Selection

**Figure 1:**
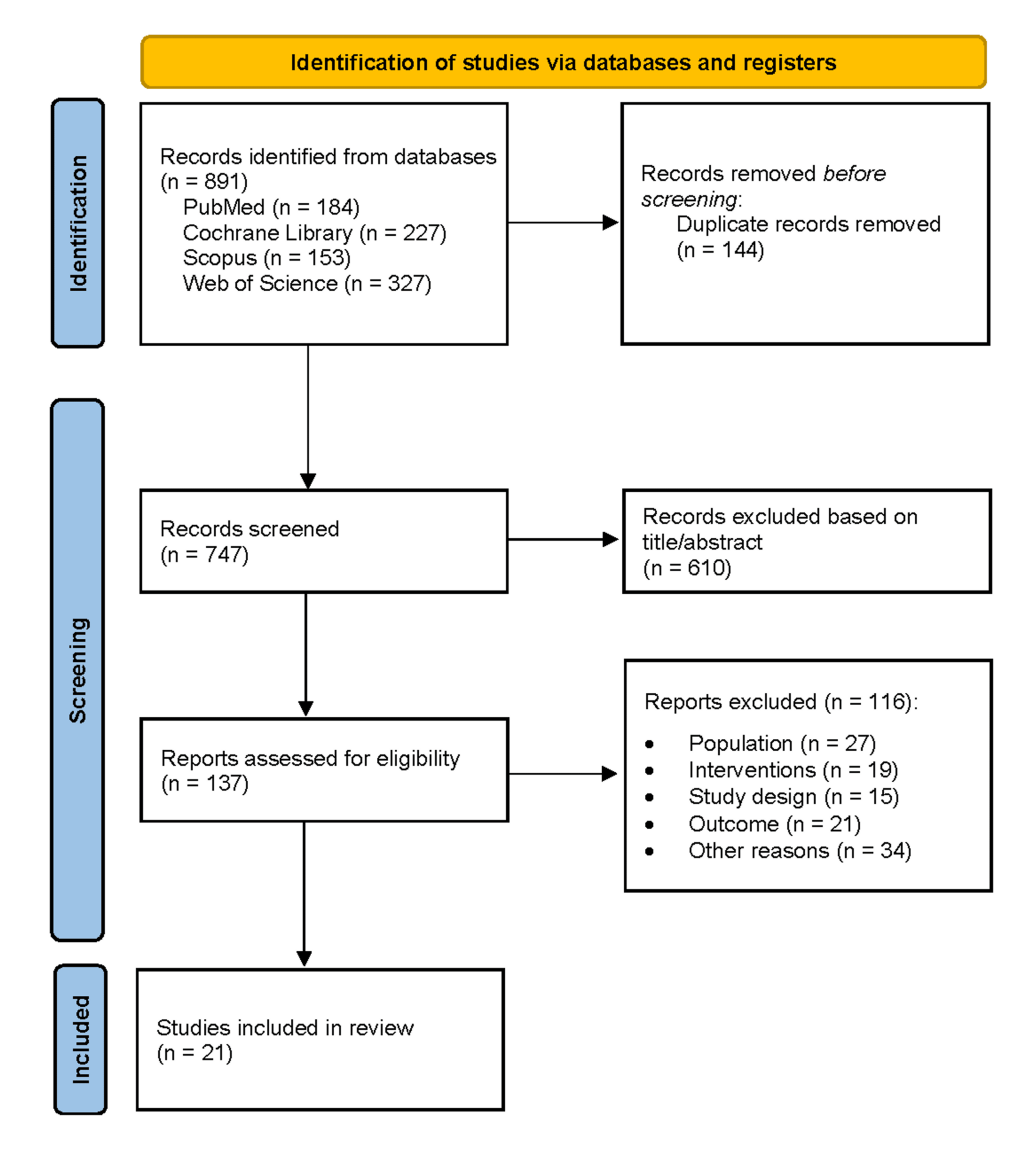
PRISMA flow diagram depicting the study selection process for the systematic review PRISMA (Preferred Reporting Items for Systematic Reviews and Meta-Analyses) flow diagram detailing the study selection process for this systematic review [22]. Following the identification of records through database searching and other sources, duplicates were removed, and the remaining records were screened. Full-text articles were assessed for eligibility, with exclusions documented along with reasons. Studies meeting the inclusion criteria were included in the final synthesis.

The search identified 891 records across four databases. After removing duplicates (n = 144), 747 records remained for screening. Of these, 610 were excluded based on title and abstract review. A total of 137 full-text articles were assessed for eligibility, with 116 excluded for reasons including study population mismatch, inappropriate interventions, non-randomized design, or irrelevant outcomes. Ultimately, 21 RCTs were included in the quantitative synthesis, along with three additional follow-up reports used for safety-related analyses (Figure 1) [1-21].

Characteristics of Included Studies

The final analysis included 21 RCTs comprising 8,392 STEMI patients undergoing PPCI. Individual trial sizes ranged from 32 to 5,115 participants. The most commonly used protocol involved four cycles of 5-minute ischemia followed by 5-minute reperfusion, typically applied to the upper limb. Timing of intervention varied across studies, with preconditioning being the most frequent approach, followed by peri- and postconditioning strategies. Follow-up duration ranged from a few days to over three years (Table 3).

**Table 3:** Study characteristics and baseline patient demographics of randomized controlled trials evaluating remote ischaemic conditioning in ST-elevation myocardial infarction undergoing primary percutaneous coronary intervention This table summarizes the methodological characteristics, intervention protocols, and baseline demographic and clinical characteristics of randomized controlled trials (RCTs) evaluating remote ischaemic conditioning (RIC) in patients with ST-elevation myocardial infarction (STEMI) undergoing primary percutaneous coronary intervention (PPCI). Data are presented for intervention (I) and control (C) groups where available. Abbreviations: RCT, randomized controlled trial; RIC, remote ischaemic conditioning; RIPC, remote ischaemic preconditioning; RIPostC, remote ischaemic postconditioning; STEMI, ST-elevation myocardial infarction; PCI, percutaneous coronary intervention; PPCI, primary percutaneous coronary intervention; SOC, standard of care; TVNS, transcutaneous vagus nerve stimulation; TIMI, thrombolysis in myocardial infarction; LAD, left anterior descending artery; LCx, left circumflex artery; RCA, right coronary artery; MI, myocardial infarction; BMI, body mass index; NR, not reported; NA, not available; d, days; m, months; y, years.

Study ID	Country	Sample size (I/C)	Intervention arm	Timing of RIC	Follow-up	Age, years (I/C)	Male, % (I/C)	Diabetes, % (I/C)	Hypertension, % (I/C)	Smoking, % (I/C)	Symptom-to-balloon time, min (I/C)	Killip class	Pre-PCI TIMI flow	Infarct-related artery
Bainey et al., 2022 [1]	Canada	129/123	RIC vs sham	Pre-/intra-procedural	1 y	63/52	76/83	26/18	54.8/30	36.5/56	209.7/197.7	≤2	NA	NA
Bøtker et al., 2010 [2]	Denmark	166/167	RIPC vs SOC	Pre-procedural	30 d	61.3/63	69.6/52.1	19.6/13	36.3/91.3	37.3/60.8	196/NA	NA	NA	NA
Chen et al., 2022 [3]	China	30/30	Repeated RIC vs control	Post-procedural	1 m	62/61	76/56	NR	38/84	56/40	209.7/NA	NA	TIMI 0–III	LAD/RCA/LCx
Crimi et al., 2013 [4]	Italy	48/48	RIC vs standard PCI	Post-procedural	12 m	63/63	75/75	NR	24/30	57/71	210.3/209.3	NA	TIMI 0–III	LAD/RCA/LCx
Eitel et al., 2015 [5]	Germany	232/232	Repeated RIC vs standard care	Pre-/intra-procedural	6 m	61.7/62	80/93	26.7/8	56.7/24	60/57	438/204.3	I–II	TIMI 0–I	LAD/LCx/RCA
Elbadawi et al., 2017 [6]	Egypt	30/30	RIC vs sham	Post-procedural	6 m	59.5/66.1	83.3/78	36.7/11	56.7/50	46.7/22	414/227	I–II	TIMI 0–I	LAD/RCA/LCx
García del Blanco et al., 2021 [7]	Spain	102/120	RIC vs sham	Pre-/intra-procedural	7 d	61/61.7	85/76	NR	54/41	42/47	182.7/241	NA	TIMI 0–I	LAD
Gaspar et al., 2018 [8]	Portugal	231/217	RIC vs control	Pre-procedural	3.7 y	56/56.3	90/54.1	15/13.5	53/54.1	54/35.1	195.3/NA	NA	TIMI 0–I	LAD/RCA/LCx
Haller et al., 2020 [9]	Austria	16/16	RIC + postconditioning vs sham	Pre-/intra-procedural	1 m	64.7/56.5	73/52.9	25/8.8	74/55.9	47/29.4	223.3/NA	I–IV	TIMI 0–III	LAD/LCx/RCA
Hausenloy et al., 2019 [10]	International	2546/2569	RIC vs sham	Pre-/intra-procedural	12 m	64.3/62.9	71/61	24/30	75/48	41/73	217/193	I–IV	NA	LAD/LCx/RCA
Ikonomidis et al., 2021 [11]	Greece	90/90	RIPostC vs sham	Post-procedural	12 m	53/61.2	83.3/60	40/30	40/43	63.3/67	NA/192	I–II	TIMI 0–III	LAD
Manchurov et al., 2014 [12]	Russia	23/25	RIC vs standard care	Pre-procedural	7 d	50.1/58.7	83.3/81.6	43.3/10.5	26.7/28.9	76.7/40.5	NA/167.2	I–II	TIMI 0–I	LAD dominant
Munk et al., 2010 [13]	Denmark	123/119	RIC vs sham	Pre-procedural	30 d	62.2/58.9	86.3/82.9	23.5/28.6	45.1/34.3	47.1/20	137/158.2	I–IV	TIMI 0–I	LAD/RCA/LCx
Prunier et al., 2014 [14]	France	18/17	RIC vs sham	Pre-procedural	3 d	60.8/59.3	81.7/94	19.2/9	47.5/17	45/45	137/150.3	I	TIMI 0–I	LAD dominant
Qian et al., 2018 [15]	China	37/34	RIC vs sham	Post-procedural	6 m	59.3/62	83/96	NR	49/28	61/30	270/163.7	I–III	TIMI 0–III	LAD/RCA/LCx
Rentoukas et al., 2010 [16]	Greece	33/30	RIC vs standard care	Pre-/intra-procedural	NR	62/68.8	77/78.8	30/31.5	49/52.1	57/61.8	244/352.3	I–III	TIMI 0–III	LAD/RCA/LCx
Vanezis et al., 2018 [17]	UK	38/35	RIC vs control	Chronic post-PCI	4 m	63.2/67.9	NA/79.1	0/32.5	75/58.3	56.2/63.2	NA/361.7	NA	TIMI 0	LAD/RCA/LCx
Verouhis et al., 2016 [18]	Sweden	47/46	RIC vs sham	Peri-/post-procedural	7 d	59.6/57	NA/90.2	6.2/17.1	37.5/39	56.2/56.1	NA/280	NA	TIMI 0–I	LAD/RCA/LCx
Wang YL et al., 2022 [19]	China	165/163	RIC vs SOC	Pre- and chronic post-PCI	12 m	63.9/59	76/88.1	11.9/14.3	43.7/52.4	40.5/71.4	195.3/266	I–IV	TIMI 0–III	LAD/RCA/LCx
Wang L et al., 2026 [20]	China	41/42	RIC + TVNS vs sham	Pre-procedural	7 d	63.1/67	77.6/76	10.4/31	40.1/61	40.9/59	194.7/326	I–II	TIMI 0–I	LAD/non-LAD
Yamanaka et al., 2015 [21]	Japan	47/47	RIC vs sham	Pre-procedural	30 d	53/67	80/76	17/37	27/65	53/51	197.3/360	≤2	NA	LAD/LCx/RCA

Baseline characteristics were generally balanced between groups. Mean age ranged from 53 to 68 years, and most cohorts were male (approximately 70-90%). Common comorbidities included hypertension, diabetes mellitus, dyslipidaemia, and smoking. The left anterior descending (LAD) artery and right coronary artery (RCA) were the most frequently affected vessels, with most patients presenting with thrombolysis in myocardial infarction (TIMI) flow grade 0 before PCI.

Risk of Bias Assessment

Overall, the included studies demonstrated low risk or some concerns according to RoB 2 assessment. The main methodological limitations were related to blinding and deviations from intended interventions in procedural settings (Table 4).

**Table 4:** Risk of bias assessment of randomized controlled trials evaluating remote ischaemic conditioning in ST-elevation myocardial infarction This table presents the risk of bias assessment of randomized controlled trials (RCTs) included in the systematic review, evaluated using the Cochrane Risk of Bias 2 (RoB 2) tool [24]. Each study is assessed across five domains: D1 (bias arising from the randomization process), D2 (bias due to deviations from intended interventions), D3 (bias due to missing outcome data), D4 (bias in measurement of the outcome), and D5 (bias in selection of the reported result). Judgements for each domain are reported using a standardized, symbol-based system: low risk of bias (+), some concerns (−), and high risk of bias (X). The overall risk of bias judgment reflects the cumulative assessment across all domains for each study. Abbreviations: RCT, randomized controlled trial; RoB 2, Cochrane Risk of Bias 2 tool; D1–D5, bias domains as defined above.

Study ID	D1	D2	D3	D4	D5	Overall
Bainey et al., 2022 [1]	+	+	-	+	-	-
Bøtker et al., 2010 [2]	+	-	X	+	-	X
Chen et al., 2022 [3]	-	-	+	+	+	-
Crimi et al., 2013 [4]	+	-	-	+	+	-
Eitel et al., 2015 [5]	+	+	-	+	+	-
Elbadawi et al., 2017 [6]	X	X	-	+	+	X
García del Blanco et al., 2021 [7]	+	-	-	+	+	-
Gaspar et al., 2018 [8]	+	-	+	-	+	-
Haller et al., 2020 [9]	+	+	-	-	+	-
Hausenloy et al., 2019 [10]	+	+	+	+	+	+
Ikonomidis et al., 2021 [11]	+	+	+	+	+	+
Manchurov et al., 2014 [12]	-	+	+	-	-	-
Munk et al., 2010 [13]	-	+	+	+	-	-
Prunier et al., 2014 [14]	+	X	X	+	+	X
Qian et al., 2018 [15]	X	-	+	-	+	X
Rentoukas et al., 2010 [16]	-	+	+	+	-	-
Vanezis et al., 2018 [17]	+	-	+	+	+	-
Verouhis et al., 2016 [18]	+	X	X	+	+	X
Wang YL et al., 2022 [19]	-	+	+	+	-	-
Wang L et al., 2026 [20]	-	-	X	+	+	X
Yamanaka et al., 2015 [21]	+	-	+	+	-	-

Primary and Secondary Outcomes

Left ventricular ejection fraction (LVEF) data were available from eight trials, including 731 patients in the RIC group and 724 in the control group. Pooled analysis demonstrated a small but statistically significant improvement in LVEF with RIC (MD 1.23%, 95% CI 0.05-2.42; p = 0.04), with no observed heterogeneity (I² = 0%).

Infarct size was reported in nine studies involving 859 RIC and 852 control patients. No significant difference was observed (MD −0.62%, 95% CI −1.61 to 0.38; p = 0.22), with low heterogeneity (I² = 7.46%).

Among secondary outcomes, no significant differences were observed for all-cause mortality, cardiovascular mortality, MACE, heart failure hospitalization, or revascularization. However, recurrent myocardial infarction was significantly reduced in the RIC group. Sensitivity analyses confirmed the robustness of most findings, although certain endpoints showed variability when large trials were excluded (Table 5).

**Table 5:** Primary and secondary clinical outcomes of remote ischaemic conditioning in patients with ST-elevation myocardial infarction undergoing primary percutaneous coronary intervention This table summarizes the pooled effects of remote ischaemic conditioning (RIC) as an adjunct to primary percutaneous coronary intervention (PPCI) in patients with ST-elevation myocardial infarction (STEMI), based on the included randomized controlled trials [1-21]. Outcomes are categorized into primary outcomes (left ventricular ejection fraction and infarct size) and secondary outcomes (all-cause mortality, cardiovascular mortality, major adverse cardiovascular events, heart failure hospitalization, recurrent myocardial infarction, and revascularization). Effect estimates are presented as mean difference (MD) for continuous outcomes and log risk ratio (log RR) for dichotomous outcomes, along with corresponding 95% confidence intervals (CI), p-values, and heterogeneity (I²). Negative values indicate a reduction in outcome in the RIC group compared with control.

Outcome	Type	No. of Studies	RIC (n)	Control (n)	Effect Measure	95% CI	p-value	I² (%)
Left ventricular ejection fraction (LVEF)	Primary	8	731	724	Mean difference = 1.23%	0.05 to 2.42	0.04	0
Infarct size		9	859	852	Mean difference = −0.62%	−1.61 to 0.38	0.22	7.46
All-cause mortality	Secondary	11	—	—	Log RR = 0.03	−0.16 to 0.22	0.75	0
Cardiovascular mortality		11	—	—	Log RR = −0.40	−0.91 to 0.11	0.13	33.1
Major adverse cardiovascular events (MACE)		9	—	—	Log RR = −0.19	−0.40 to 0.01	0.06	34.67
Heart failure hospitalization		11	—	—	Log RR = 0.18	−0.48 to 0.11	0.23	19.56
Recurrent myocardial infarction		14	—	—	Log RR = −0.24	−0.48 to −0.01	0.04	0
Revascularization		10	—	—	Log RR = 0.13	−0.07 to 0.34	0.19	0

Sensitivity Analyses

Leave-one-out analyses confirmed robustness for most outcomes. Mortality, infarct size, LVEF, and revascularisation remained stable. Cardiovascular mortality, MACE, and HF hospitalisation showed partial sensitivity to large trials. Recurrent MI remained consistently significant.

Discussion

The findings of this systematic review demonstrate a consistent but nuanced pattern across trials evaluating RIC in STEMI patients undergoing PPCI. While early and smaller randomized trials suggested beneficial effects on surrogate markers of myocardial injury, larger and more contemporary studies have generally failed to confirm a significant impact on major clinical outcomes. This divergence reflects both biological complexity and substantial heterogeneity in study design, intervention protocols, and patient populations.

Several early and single-center trials reported improvements in surrogate endpoints such as infarct size, myocardial salvage, and left ventricular function [2,13,16]. These findings support the mechanistic rationale of RIC, which is thought to attenuate ischaemia-reperfusion injury through systemic protective signalling pathways. Importantly, studies in which RIC was initiated early, particularly in the pre-hospital or pre-procedural setting, tended to demonstrate more pronounced effects, suggesting that timing is a critical determinant of efficacy [2,13]. In contrast, trials applying RIC after reperfusion or in delayed repeated protocols showed less consistent benefit, indicating that the therapeutic window for cardioprotection may be limited.

However, these favorable signals were not consistently replicated in larger multicenter trials. The CONDI-2/ERIC-PPCI trial [10], which represents the largest study included in this analysis, found no reduction in cardiac death or heart failure hospitalization despite adequate power and rigorous methodology. Given its substantial sample size, this trial heavily influenced pooled estimates and highlights the challenge of translating improvements in surrogate markers into meaningful clinical benefit. Similarly, other contemporary trials with standardized PPCI protocols and optimized medical therapy have reported neutral effects on infarct size and functional recovery [5,8].

Several factors may explain the discrepancy between early positive studies and later neutral findings. First, advances in STEMI management-including shorter ischaemic times, improved antithrombotic therapy, and routine use of guideline-directed medical therapy-may have reduced the incremental benefit achievable with adjunctive interventions such as RIC. In this context, the relative contribution of ischaemia-reperfusion injury to overall myocardial damage may be smaller, thereby limiting the detectable effect of cardioprotective strategies.

Second, there was considerable heterogeneity in RIC protocols across studies, including differences in limb selection, number of cycles, inflation pressure, duration, and timing relative to PPCI. For example, some trials employed upper limb conditioning with four cycles of 5-minute inflation/deflation, whereas others used lower limb protocols or varied cycle durations [4,8]. Chronic or repeated RIC strategies, such as in Chen et al. [3], introduced additional variability by targeting post-infarction remodeling rather than acute injury, which may explain their differential impact on functional outcomes without clear effects on infarct size.

Third, co-interventions may have influenced outcomes. Some trials evaluated RIC in combination with other therapies, such as pharmacological agents or neuromodulation strategies. The study by Wang L et al. [20], which combined RIC with transcutaneous vagus nerve stimulation, demonstrated improved cardiac function and reduced reperfusion injury markers compared to control, suggesting potential synergistic effects. However, such multicomponent interventions complicate attribution of benefit specifically to RIC and introduce challenges for inclusion within a homogeneous meta-analysis framework.

Another important consideration is the reliance on surrogate endpoints. While modest improvements in left ventricular function were observed in pooled analyses, the clinical significance of these changes remains uncertain. Small absolute differences in LVEF may not translate into reductions in mortality or heart failure, particularly in well-treated contemporary populations. This disconnect between surrogate and clinical outcomes has been a recurring theme across cardioprotection trials.

Despite these limitations, a consistent finding across the included studies is the safety and feasibility of RIC. The intervention is non-invasive, low-cost, and easily applicable in both pre-hospital and in-hospital settings. Moreover, the observed reduction in recurrent MI in the pooled analysis suggests that RIC may exert systemic vascular or endothelial effects beyond the acute infarct phase, warranting further investigation.

Overall, the current body of evidence suggests that while RIC has a strong mechanistic basis and modest effects on surrogate markers, its impact on clinically meaningful outcomes remains uncertain. Future studies should focus on identifying patient subgroups most likely to benefit, standardizing RIC protocols, and exploring combination strategies that may enhance cardioprotective signaling.

## Conclusions

Remote ischaemic conditioning as an adjunct to PPCI in STEMI shows modest improvement in left ventricular function and reduction in recurrent myocardial infarction. However, it does not demonstrate clear benefits in mortality, MACE, heart failure hospitalisation, or revascularisation. Overall, while biologically plausible and potentially beneficial in selected outcomes, current evidence does not support routine clinical implementation. Further large-scale, standardized RCTs are required to define its role and identify responsive patient subgroups.
